# Raising a beautiful swan: a phenomenological-hermeneutic interpretation of health professionals’ experiences of participating in a mealtime intervention inspired by Protected Mealtimes

**DOI:** 10.1080/17482631.2017.1360699

**Published:** 2017-08-23

**Authors:** Malene Beck, Bente Martinsen, Regner Birkelund, Ingrid Poulsen

**Affiliations:** ^a^ Institute of Public Health, Department of Nursing Science, Aarhus University, Copenhagen, Denmark; ^b^ Department of Neurology, Zealand University Hospital, Roskilde, Denmark; ^c^ Institute of Health, Department of Nursing Science, Aarhus University, Copenhagen, Denmark; ^d^ Section of Health Services Research Lillebaelt Hospital, University of Southern Denmark, Odense, Denmark; ^e^ Institute of Public Health, Aarhus University Research Manager at the Research Unit on Brain Injury Rehabilitation Copenhagen (RUBRIC), Clinic of Neurorehabilitation, TBI Unit, Rigshospitalet, Hvidovre, Denmark

**Keywords:** Mealtimes, interventions, nursing, protected mealtimes, qualitative studies, phenomenology-hermenutic, Paul Ricouer

## Abstract

The British concept named Protected Mealtimes is known for stopping all non-acute activities and giving health professionals an opportunity to focus on providing patients their meals without being interrupted or disturbed. PM involves a cultural and behavioural change in the clinical setting, since health professionals are asked to adjust their daily routines. This study investigate how health professionals experience participating in a mealtime intervention inspired by the concept of Protected Mealtimes and intend to change mealtime practices. Three focus group interviews was conducted and included a total of 15 interdisciplinary staff members. After transcribing the interviews, the text material was analysed and interpreted in a three-methodological-step process inspired by the French philosopher Paul Ricoeur.

In the analysis and interpretation three themes was identified. The themes were: (1) a chance towards a new and better scene; (2) a step towards a more neurologically friendly environment; and (3) a renewed view of the neurological patients. This study concludes that to the health professionals, the intervention was meaningful in several ways because it created structure during mealtimes and emphasized the importance of creating a calm environment for both patients and health professionals. The intervention was described as an eye-opening and well-regarded event in the field of neurological care that facilitated community, and reflections on nursing care and professional identity were expressed.

## Introduction

In *The Ugly Duckling*, a famous fairytale written by the Danish writer H.C. Andersen (Andersen, 2016), a young swan is hatched on a duck farm. In the first part of the story, the young swan does not know it is a swan, and neither do the other animals on the farm. Because the swan does not look like a duck, it is not respected by the other animals on the farm. Not, at least, until the end of the story, when Andersen reveals that the ugly duck is, in fact, a beautiful swan. The young swan realizes its identity after bowing its head and seeing its reflection in the lake. Andersen’s fairytale exemplifies this present study, because it elaborates how enlightenment can provide the health professionals, especially nurses, a renewed view on identity and internal fortitude. This study presents how health professionals, and especially the nurses, realize the importance of creating a calm environment for both patients and themselves through participating in an intervention inspired by Protected Mealtimes (PM) and intended to change mealtime practices.

## Background

PM is an established initiative characterized by stopping all non-acute activities during mealtimes. The aim of PM is to focus on the patients’ mealtimes by assisting the patients while eating and facilitating an environment without interruptions or disturbing activity. PM is a well-known concept, but little research examining the phenomenon has been conducted.

In the existing literature, PM has often been effective in changing clinical practice. Studies indicate that PM has resulted in staff members being more involved and engaged in mealtime-related care (Dickinson & Welch, 2006). Nurses prioritized their time differently, had fewer distractions, and provided more assistance to patients at mealtimes when PM was implemented in clinical practice (Chan & Carpenter, 2015; Dickinson & Welch, 2006; Ullrich et al., 2008). However, conflicting results exist regarding the effect of PM on patients. Some studies have suggested that even though patients valued the protected mealtimes, their food intake did not increase (Hickson, Connolly, & Whelan, 2011; Young et al., 2016) and energy and protein consumption did not change (Huxtable & Palmer, 2013). However, the implementation of PM is justified in the research literature by claiming that some aspects of the PM concept showed significant improvement, for example increased mealtime assistance (Porter, Ottrey, & Huggins, 2017), as well as increased mealtime interruptions. Furthermore, researchers identified that PM contributes positively to the mealtime environment, even if the quality of the concept cannot be quantified.

Given that there has been little research conducted in this area, and evaluations of PM programmes in the existing literature have shown mixed results, further research needs to be conducted. The PM concept involves a cultural and behavioural change in relation to mealtimes, since the health professionals are asked to adjust their daily routines when serving meals. Therefore, the health professionals’ perspectives on PM and their experiences of being a part of an intervention are important to obtain in-depth insight into the phenomenon of PM and knowledge about the experiences of participating in changing clinical practice. The health professionals’ perspectives may help nuance the discussion of how PM affects not only the patients’ nutritional intake but also staff members’ professional lives and perspectives on mealtime care. Thus, a qualitative evaluation of the PM programme from the perspective of health professionals can inform future interventions focusing on positively changing mealtime practices.

## Aim

The aim of the study was to investigate how health professionals experience participating in a mealtime intervention inspired by the concept of Protected Mealtimes and intended to change mealtime practices.

## Method

### Design

To study the professionals’ perspectives of being a part of an intervention guided by the PM concept we used a qualitative approach consisting of focus-group interviews inspired by the methods described by Halkier (2002). These interviews were conducted in order to let the professionals disclose their experiences of participating in dynamic discussions, where they were given the opportunities to elaborate on this together using their own concepts and language (Halkier, 2002). This design was well suited to help shed light on the pathways through which an intervention generates its impacts, i.e., why it was successful, how the intervention worked and how it could be optimized (Ludvigsen et al., 2013). Hence, the design also worked as a guidance in relation to the organization, because the management of the department evaluated the benefits of the intervention based on the findings from this study.

The interviews were analysed and interpreted using a phenomenological-hermeneutic approach inspired by the French philosopher Paul Ricoeur (1979). Obtained through focus-group interviews, the health professionals’ narratives were used to elucidate their experiences, perceptions, and the intentions of their actions during the intervention (Dreyer & Pedersen, 2009; Pedersen, 1999). In accordance with the philosophies of Ricouer, the narratives obtained through the focus-group interviews revealed the participants’ (professional) life worlds in the context of the intervention (Ricouer, 1984). By using this approach it became possible to investigate in-depth something new of being-in-the-world as a professional during an intervention changing traditional settings. Ricouer (1984) states that there is a surplus of meaning in a text which requires a process of interpretation and understanding in order to gain new insight into a phenomenon.

### Framework of the intervention

The framework for developing and evaluating complex interventions proposed by the British Medical Research Counsel (MRC) (Craig et al., 2008a, 2008b) was applied. According to the MRC, the development of complex interventions consists of several phases, key elements of which include a thorough literature search, inclusion of relevant theory, and (pilot) testing the intervention. Furthermore, continuous evaluation and adjustment are important when testing interventions and creating scientific knowledge regarding the application of these interventions in clinical practice (Craig et al., 2008a, 2008b).

This study maintained an intervention called *Quiet Please*. The overall goal of this intervention was to change mealtime practices by creating an environment inspired by PM. The international research literature described a number of factors that must be considered when introducing interventions in clinical environments to prepare the staff to implement changes in practice. Changing practices in this setting is a complex process that has proven to be challenging because the routines and attitudes that are part of a hospital’s organizational culture are often not easy to change (Copnell & Bruni, 2006). However, research suggests that the involvement of staff has a positive effect on the implementation of the interventions in practice (Dickinson & Welch, 2006; Ullrich, McCutcheon, & Parker, 2011). Because motivation can be difficult to establish, McCormack, Manley, Kitson, Titchen, & Harvey (1999) emphasized the importance of internal facilitators during practice development.

### Conducting the intervention

A literature search, the theory of basic nursing, and empirical studies (Beck, Martinsen, Poulsen, & Birkelund, 2016) informed the creation of the intervention *Quiet Please*. An important part of the intervention was to develop and test a mealtime policy in clinical practice. The policy was inspired by PM, but was modified for the Danish and neurological care contexts.

In this study, health professionals were involved from the beginning. This was accomplished by inviting the nurse responsible for nutritional care together with the first author on a site visit to Cambridge University Hospital to observe how PM was performed. The inclusion of this nurse demonstrated feasibility in clinical practice and enabled the nursing staff to have ownership of the project (Hallpike, 2008). Furthermore, a working group, consisting of nurses from different departments with varied seniority levels and areas of specialized knowledge, was established. The purpose of the working group was to establish role models during the intervention (McCormack, Manley, Kitson, Titchen, & Harvey, 1999). The working group also contributed to the intervention with the knowledge of the context, practices, and culture of the department so that potential barriers to the intervention could be confronted.

To involve all clinical staff members in the intervention, all nurses were taught the principles of PM. This instruction consisted of a mandatory one-hour lesson focused on mealtime and nursing care (Dickinson, Welch, & Ager, 2008; Ullrich et al., 2011). The teaching courses continued for 3 months (August– November 2016) and ended by inviting all staff members to a “Kick-off” day, during which invited speakers presented the different mealtime components, and the new mealtime policy *Quiet Please* was presented again.

Based on earlier findings (Beck et al., 2016) that had shown a lack of aesthetic elements in the mealtime environment, it was decided to include menus, flowers, napkins, and other cans to see how this was assigned meaning. The process of tailoring the policy was completed by translating the British policy (obtained during the site visit to Cambridge) and adding significant recommendations related to patient perspectives. The policy was adjusted to fit the local context; consequently, it was presented to and adjusted according to advice from the working group and subsequently approved by the management. The policy was implemented in the department in November 2014.

### Participants

With the aim of providing insight into meal-related perceptions, interactions, and norms that existed among health professionals during the intervention period, 15 staff members were selected to participate in focus-group interviews (Halkier, 2002; Morgan, 1996). The selection of participants was conducted in cooperation with the ward management, who aided in determining the degree to which the different participants were exposed to the intervention (e.g., lower exposure due to working nightshifts or vacation) (Malterud, 2011). To obtain variation, the health professionals selected included a range of different participants in terms of age, education, seniority, and experience within rehabilitation and whether neurology was a specialized focus (Halkier, 2002).

### Data collection

Data were collected in the setting of a department of neurology. In this study, three focus groups with five participants each were conducted to enable processing the data in depth and uncovering staff attitudes towards meals and meal routines in the context of the intervention implemented in the department. Two of the focus group consisted of nurses from each ward, and one focus group included other interdisciplinary staff, including a social worker, secretary, and physical, occupational, and speech therapists. The focus group interview was planned with a semi-structured interview guide based on the theory of the mixed “funnel” model (2002). The interview was launched with open exploratory questions, where participants themselves were allowed to choose what and how they talk about the subject within a broad framework. Here, there was room for observing group dynamics and generating knowledge through the group (Halkier, 2002). As the interview progressed, a “tighter” control toke place, where the moderators became more specific in their questions, thus ensuring that the group was highlighting the areas of interest. The purpose of the interview guide was therefore two-fold: first, to get the group to discuss the topics contained in the guide—and partly that the same questions focusing on the intervention were asked in all three interviews. Because the first author had been the facilitator during the intervention, two of the supervisors and co-authors conducted the interviews and worked as moderators. Their roles during the interviews were to encourage the health professionals to talk about the meals and meal structures and manage the social dynamics that took place during the conversation. They focused on listening and being open and appreciative while ensuring that all the health professionals had the opportunity to speak and be heard. This was accomplished, for example, by asking the health professionals who had not spoken, “What do you think about what has been said?”, to invite them to participate in the interview (Halkier, 2002). The interviews were conducted in a room that was suitable for interviewing purposes, in which staff could talk about their experiences in safe and undisturbed surroundings. Moreover, the room contained the different tools included in the intervention including aesthetic elements such as napkins, a special can for milk, and sauce. Each session lasted approximately 1½ hours, as this left out space for both the introduction, the interview itself, and the outro of the interviews (Halkier, 2002).

### Data analysis and interpretation

The interviews were transcribed and the text material was analysed and interpreted using a phenomenological-hermeneutic approach inspired by Paul Ricoeur’s philosophy on narratives and interpretation (Ricoeur, 1976, 1979). The focus-group interviews were phenomenological in that they had a descriptive nature but also included a hermeneutic element through the inclusion of narrative interpretation (Ricoeur, 1976). It was the health professionals’ narratives that were analysed and interpreted because, according to Ricouer, the narrative, in and of itself, has the potential to provide knowledge about the factors affecting humanity (Ricoeur, 1973, 1976, 1979).

Ricouer (1979) cemented the connection between phenomenology and hermeneutics and points out the mutual affinity between them (Tan, Wilson, Olver, & Barton, 2011). Given Ricouer’s notion, interpretation can be considered as a key element when performing phenomenological-hermeneutic research, because description and explanation alone are not sufficient as regards obtaining an in-depth understanding of the experiences related to human existence. In Ricouer’s view, hermeneutics is text-oriented interpretation, where an effort towards cognition, in order to interpret and search for the surplus of meaning that is stored in the human life world, is sought (Ricouer 1976). Fundamental to Ricouer’s theory of interpretation is his understanding of text and, in particular, his concept of distanciation, a standing separate from and objectifying of the text (Tan et al., 2011). This means that a Ricoeur-inspired analysis can facilitate interpretation of meaningful aspects in the empirical material collected through the interviews. Ricoeur (1979) argues that when the discourse is recorded in writing, it creates a distance that frees the meaning from the event and the author, whereby an interpretation option opens. Through this approach, a movement from a surface interpretation to an in-depth interpretation of the transcribed empirical material is obtained. Ricouer avers that: “A text’s importance is not behind the text, but in front of it” (1979, p. 214) and “to understand a text is to follow its movement from sense to reference, from what it says to what it talks about” (1979, p. 214), thus revealing the text’s case and reach from what the text says to what it talks about. A structural analysis is a central intermediary link between the initial interpretation and the in-depth interpretation. Hence, it can be considered as the explanatory torque that helps understanding. Thus, there is no contradiction between explanation and understanding, but instead the explanation is used in service of understanding the importance, and the meaning of the text is identified throughout interpretation (Dreyer & Pedersen, 2009). The following three methodological phases were used when data were analysed and interpreted, and are presented more systematically than they were actually performed, due to the dialectical movement (Ricoeur, 1979).

While striving to understand the health professionals’ experiences, we employed a three-phased interpretation, including an *interpretation, structural analysis*, and *comprehensive understanding and discussion* (Lindseth & Norberg, 2004). *The naive reading* was conducted by reading the material through several times to achieve an initial understanding through a naive interpretation. This was followed by a *structural analysis*, which was a necessary stage between the naive interpretation and comprehensive understanding. In this phase, the text was structured openly into meaningful units, from which themes or subthemes where gathered. These were compared with the findings of the naive reading, and the guesses that were derived at this level. The purpose of the structural analysis was to derive a possible explanation and understanding of “what the text said” towards “what it speaks about”. Moving the text from the specific to the general (Dreyer & Pedersen, 2009), the themes were further discussed and interpreted in the context of relevant theory and empirical studies. The last phase, *comprehensive understanding*, is the dialectical process between explanation and understanding in which the movement from the initial interpretation towards a more comprehensive understanding can be considered as an endless spiral whereby new dimensions and meanings are created.

### Ethical considerations

All participants received written and verbal information about the project. Participation took place under informed consent. Participants were reminded that they could, at any time, withdraw from the interview without justification of any kind. Participants were also informed that their responses would be anonymized. As the interviews were conducted by the project’s supervisors, who were unfamiliar to the participants, the participants had the opportunity to speak openly about the experiences during the intervention. This project has been approved by the Danish Data Projection Agency and implemented under the framework of the umbrella agreement on data processing, which is applicable to Region Sealand’s Health Care and in line with international ethical guidelines (Nordic Nurses Federation, 2003).

### Theoretical perspectives

Ricouer’s theory on narratives was applied to the themes identified in the structural analyses (Ricoeur, 1984). Ricouer’s theory on narratives can help elucidate the importance of the narrative and storytelling in human life. According to Ricouer, narratives can be understood as a way of expressing action. Ricouer writes, “To understand a story is to understand both the language of ‘doing something’ and the cultural tradition from which proceeds the typology of plots” (Ricouer, 1984, p. 57). This means that the role of the narrative is twofold: it can create meaning but also articulate meaning. Hence, the narrative can be considered both as an articulation of an already known world and as a structure used to understand and explain the world (Ricoeur, 1984). In other words, listening to narratives not only elaborates experience but also means adapting the structure of the experience, and narrative competences are developed when telling the story again (Horsdahl, 1999). Hence, the narrative is powerful by its nature because it connects the unique with the general (Ricoeur, 1984), and identity can be created by the recognition and explanation of the interpretations that narratives maintain (Kemp, 1990; Ricoeur, 1984).

## Findings

### Naive reading

The initial impression of the material was that the health professionals experienced participating in changing mealtime practices as a meaningful process because it added valuable elements (e.g., emphasizing the importance of calmness) to the mealtime activities. In the naive reading, it was identified that changing mealtime practices had been an eye-opening experience for the health professionals and that positive aspects of the mealtimes had been enhanced. However, the material also left the impression that the positive perceptions of *Quiet Please* were dependent upon a logic of efficiency and rationalism, which could challenge the considered value of the mealtime activity.

## Structural analysis

### A change towards a new and better scene

During the interviews, the health professionals emphasized that there was room for improvement in the traditional mealtime setting. In all the interviews, it was expressed that the mealtime environments were in particular need of change:

Before (*Quiet Please*) it was such a boring experience and a somewhat lonely experience for the patients. They were sitting in the hallway or most of them were sitting in their bed and ate next to their tables (FG1). Mealtimes were chaotic and were something that you had to hurry so you could get it over with (FG2). I had the feeling that it (the mealtimes) was about getting it over with because the person in charge, either nursing staff or cleaning staff, had to get it out and then pack it together again (FG1).

As illustrated in the quotations, the traditional mealtimes were described as an activity that had low priority because it occurred in combination with tasks that were the responsibility of cleaning staff. Furthermore, it was described as an activity that had to be completed while handling other tasks. This also suggested that the traditional mealtimes were not considered as an integrated part of the patients’ care and rehabilitation. In fact, the health professionals described the traditional mealtime as an activity that had no quality in terms of both the patients’ experiences and their care and rehabilitation. Using words such as lonely, loud, messy, chaotic, hectic, and interrupted emphasized the need for improvement and suggested that the health professionals lacked ownership of the meals as a part of their daily care for the patients. Mealtimes were not an activity that had been considered as a natural element in the patient’s rehabilitation process.

Introducing *Quiet Please* changed the health professional’s perspectives of mealtimes. This new perspective meant that the health professionals considered mealtimes as beneficial to both the patients and themselves. A nurse says:

I remember we had some young patients, and even though they were very different because of their diagnosis and their situation, they suddenly sat together, four men and women, and found each other in the hallway. They ate together. It was so interesting to see how the patient with aphasia, who could not speak, became actively involved in the fun around the table. The others were interested in him and asked him stuff and he tried his best to tell them. Therefore, there was also a lot of social benefit (*Quiet Please*) (FG3).

As suggested by the quotation, the health professionals had felt that *Quiet Please* created togetherness among the patients because they had something they could share. Togetherness was identified in the relationship between the health professionals and the patients. In particular, the nurses described how *Quiet Please* became an important relational practice because it was a way of being closer to the patients. This finding was important because *Quiet Please* according to the nurses became a path in the clinical morass they occupied in which it was approved to just be with the patients. A nurse illustrates this by saying:

Now, I come out and see my patients—at least for a half-hour. “How do you look right now?” and “How are you?” and there comes that peace where I have the opportunity to just be with the patient … just for a little while (FG2).

It was clear from health professionals’ discussions that they regarded *Quiet Please* as an opportunity to be closer to the patients. This opportunity was described as having a physical component because, according to the nurses, it involved observations of the patient’s disabilities and physical impairments. Therefore, the professional’s closeness to the patients was related to a physical proximity because the nurses described how *Quiet Please* literally allowed them to get out of the office and next to their patient. The nurses’ description of getting back to the patients almost sounded as if they had been gone for a while and their rhetoric was comparable to that of a person describing travel and how it felt to return home. In this case, the “travelling” related to a movement away from basic nursing. This became clear when the health professionals, and especially the nurses, described how *Quiet Please* exhibited the important elements of basic nursing. The importance of *Quiet Please* was recognized and illustrated in the following quotation:

I think it (*Quiet Please*) helps to keep the focus where it needs to be. When you are educated, there has never been any doubt about where the nurse should be; she must help to ensure that patients receive proper nutrition. It (*Quiet Please*) has clearly helped to change the focus of the nurses, and I believe that this project has helped us to implement some kind of process (FG2).

During the interviews the nurses used words such as “basic nursing” or referred to Florence Nightingale when talking about *Quiet Please*. A nurse said:

You have a better feeling now when handling the mealtimes. In the past you could be a little embarrassed about the mealtimes. I also believe that the patients can feel that in us because we may present the food in a different way than before. The motivation is different now. “See what I have here” instead of “here you go, here’s lunch” (FG3).

### A step towards a more adjusted and neurologically friendly environment

The material showed that all agreed on one thing: introducing *Quiet Please* was like a drop in the sea that became little ripples in the water. This meant that introducing calmness was an activity that affected the whole environment in the department and not solely the eating environment. The health professionals often spoke of *Quiet Please*. For example, one professional said: “It is contagious” (FG2). Another says: The silence “spreads” and people walk carefully down the hallway (FG1). When asked what was spreading among the health professionals, they described it as the peace that *Quiet Please* provided. “There is this peace now” (FG1) a health professional said. The word peace was relative to the traditional mealtime setting that was considered hectic and chaotic. The peace was described as a “game-changer” by the health professionals because it was a new and unexpected activity that changed the environment in a positive way. This was illustrated when a nurse enthusiastically said:

*Quiet Please* has done something good for us. We are scaling down a bit, so we get a rest in our heads. It is healthy for us. We are handling multiple tasks at the same time, and you get enormously tired of that. And you don’t manage any more tasks anyway because then you just get forgetful instead (FG2). There is that half hour where nothing is going on, but where you simply can be allowed to be in peace and get … (break) that inner peace (FG3).

As illustrated in this quotation, *Quiet Please* was described by the health professionals as a positive event because it provided an opportunity to shift gears and slow things down. This meant that *Quiet Please* allowed the health professionals to interrupt the fast-paced environment, think, and then act upon their thoughts. This process was described by the health professionals as a reflection period that resulted in perspectives on the daily rhythms, patient care, and on the nurses themselves. The reflection was described as a daily perk and a healthy element during the day.

In relation to the calmness that was “spreading,” the interview data suggested that not only was the calmness “contagious” but the aesthetic element was as well. One example of this occurred when the health professionals described how a folded napkin could affect the mealtime environment not only for the current and upcoming meals. The health professionals explained:

I have seen some of the nurses fold napkins into large flowers and peacocks and whatever … I think it is great. I also believe that the patients have been much more focused by this (FG2). One day I walked into a two-person room where they (the patients) had put a tablecloth on the table and sat there with flowers on the table. (FG1). Actually, I had a patient who had made her bed and cleaned the room because the mealtime was coming up. That is quite fascinating, right? The patients have actually taken this into their hearts (FG3).

As illustrated in these quotations, the health professionals described how the aesthetic elements in *Quiet Please* had spread like ripples in the water when the programme was introduced in the department. This also meant that the nurses had to change some of their mealtime routines because the patients were requesting alternatives to the traditional mealtime. This was described when the health professionals explained how the experienced patients had a more active role during *Quiet Please* than in the traditional setting. In the traditional setting, the role of the patients was described by the health professionals as passive because the patients often ate in their beds alone. Therefore, implementing *Quiet Please*, which meant putting menus and napkins on the tables, became a statement to the patients. This statement was a way of saying, “We care about your meals”, which the patients responded to by involving themselves more in the mealtimes. This meant that the patients’ individual needs and wishes, such as food choice, were respected during the mealtimes. The nurses described this as a “new” way of doing things and said:

Earlier, we did not go around to the patients in the same way and presented the menu to them as we do now. Now we have the menus and in fact, it is a part of the project that they (the patients) get to decide for themselves. Earlier, we said, “Well, you are getting the hospital diet” or “You need the normal diet”, and then there was not so much beating around the bush unless they rejected (the food) and said, “I cannot stand this”. Then we were negotiable. Now, we do it in a new way and talk to the patients. We talk about how their appetite is today.

As revealed in the quotations, *Quiet Please* was described as an element that changed the balance of who was in charge of the mealtimes. The health professionals explained that, in the traditional setting, they often decided when, where, and what the patient should eat. *Quiet Please* changed that and provided the patients with a chance to be actively involved. This was not without importance because that perspective seemed to be “spreading” among the health professionals and made them consider the mealtimes as a patient-centred activity.

### A renewed view of the neurological patients

In the interviews, the health professionals described how they considered *Quiet Please* an important event because the intervention made care more patient-centred and their professional perceptions were sharpened. However, the interviews also revealed a tension between the health professionals. The interviews indicated that the nurses considered that groups within the staff had more authority than others in prioritizing some tasks. This became clear when they repeatedly talked about how their colleagues had taken it (*Quiet Please*) on and they were glad that *Quiet Please* had been respected. A nurse said:

I think that the doctors have had to adjust some limits. Food is important for the patients. They (the doctors) have sometimes expressed that this is not their concern. I do not agree with that. Food is an important thing for people to thrive (FG2).

The quotation illustrates that while the nurses considered mealtime as a primary element in the patients’ “being”, they also considered the responsibility for the mealtimes to be their own. This meant that involving other health professionals (e.g., a doctor) in the mealtime structure and expounding upon different health professionals’ perspectives was an unnatural thought. Therefore, taking the time for meals could arouse some concern, especially in the nurses, because they collaborated to support the idea of *Quiet Please*. The doctors were not the only concern of the nurses during *Quiet Please*. Organizational structures resulted in *Quiet Please* conflicting with other tasks (e.g., cleaning) that were performed at the same time as meals were served. A nurse says about this:

We intend to use all the goodness created by this project and continue. Well, there have begun to be some issues, i.e., the cleaning staff seemed to have problems to achieve their tasks because we were making peace and calmness. They (the cleaning staff) were stressed that they could not just snatch a few bathrooms while the patients were eating (FG2).

The quotation suggests that organizational structures made it difficult to maintain calmness during mealtimes because other tasks, such as cleaning the toilets, were completed while the patients were eating. The quotation thereby gives an example of how the mealtime activities had a low place in the hierarchy of tasks that need special attention. This also meant that facilitating mealtimes was a task that, before *Quiet Please*, was not that popular, simply because it had low professional value compared to other tasks (e.g., participating in doctors’ rounds). This was in contrast with the nurses’ descriptions of their “new” role during *Quiet Please* in which renewed focus on the meal changed the hierarchy of the activity and made it an important event. Thereby, the role of the nurses also changed, and they became “gate-keepers” in the facilitation of calmness during mealtimes.

Regarding the role of the nurses, it was identified that the nurses described their roles in the hospital as if they were soldiers battling at war. This also meant that they used rhetoric that was associated with war when describing the way in which they facilitated calmness. For example, a nurse said: “We have been hit [with increased caseloads] lately and understaffed. This made it [facilitating *Quiet Please*] difficult. Some days it’s just a matter of ‘surviving’” (FG2). War rhetoric was not the only theme arising from the nurses’ descriptions of *Quiet Please*. The interviews also revealed that even though the health professionals saw *Quiet Please* as a positive, beneficial, and needed change, there was still an underlying tone of rationalism and efficiency when talking about the care and treatment of the patients. One nurse said the following:

One day I asked a student to bring me a weight and she did. Then somebody said “Hey … we are doing ‘*Quiet Please*’”. Well, although we have done dramatically better at this, you sometimes forget and want to use the waiting time until the food comes. We all had to work (to overcome) old, bad routines and focus on (the fact) that we only needed to be quiet for half an hour … but we are so rational and think “I’ll just have to manage to do this and that” (FG2).

Using war rhetoric and emphasizing the logic of rationalism and efficiency paints a picture of a battle between professionalism and organizational practice. This suggests that, according to the health professionals, interventions such as *Quiet Please* depend on daily and continual reminders of the necessity and importance of the initiative. This finding was important because it showed that if interventions such as *Quiet Please* were not supported by meaningful arguments they risked being slaughtered in the making. The health professionals underlined this by saying:

It (talking about interventions) requires that there is a driving force because in the beginning one might think, “Well, is that really necessary?” It means a lot that you have both a primary motivator and a sparring partner, one who likes to follow up on things. I think this really is a prerequisite … It IS a prerequisite for such things (FG3).

Our study showed that to the health professionals how an intervention is presented and launched was assigned great importance. The health professionals connected the meaningfulness of the intervention to the way it was introduced. The health professionals described it in these words.

We (the health professionals and the project leader) were always in a close dialogue and the way things were presented and launched meant something (FG1). For example it was an eye-opener to see the quotes from the patients that she presented to us (FG2).

## Comprehensive understanding and discussion

Our findings showed that *Quiet Please* was evaluated positively by the health professionals, because it increased the quality of the mealtime activity and made the health professionals focus more on giving patients positive (mealtime) experiences. *Quiet Please* was not only a way to create a calm environment during mealtimes in general but also a way to re-introduce health professionals, and in particular nurses, to the foundations and core principles of nursing. This initiative provided the nurses with a feeling of getting back to basics, which was not without importance because it facilitated a motivation and pride in mealtime-related care. However, this finding also indicated that the traditional hospital routines were structured without the incorporation of planned official breaks for the nurses to reflect. This is an important finding because the health professionals, and especially the nurses, argued for the necessity of calmness because a continuously hectic environment is thought of as unhealthy and not a part of the healing environment considered to be ideal for patient rehabilitation.

Mealtimes are important events because patients can experience (multiple) disabilities when eating due to their neurological disease (Carlsson, Ehrenberg, & Ehnfors, 2004; Hafsteinsdóttir, Mosselman, Schoneveld, Riedstra, & Kruitwagen, 2010; Westergren, Ohlsson, & Hallberg, 2001). Our study supports the importance of mealtimes in neurological settings and shows that there was room for setting a new and better scene. This is in line with earlier studies that emphasized the necessity of changing mealtime practices because mealtime care was considered to have low priority and status compared with other tasks by caregivers (Martinsen & Norlyk, 2012; Ullrich et al., 2011). Similarly, our study identifies that in traditional settings, mealtime care before the intervention was not considered naturally integrated into care and rehabilitation, even though mealtime care has been recognized as an important element in the treatment of patients with neurological diseases (Jefferies, Johnson, & Ravens, 2011; Martinsen, 2005). The concept of Protected Mealtimes was considered by Dickinson et al. (2008) to be an important tool for changing mealtime practices in which patients could eat with interruptions. This contrasts with the findings by Huxtable and Palmer (2013) that suggested that interruptions by the caregivers were increased by providing Protected Mealtimes. However, our study showed, in line with Ullrich et al. (2011), that mealtime interventions inspired by Protected Mealtimes are not only about decreasing interruption during mealtimes, but also about providing ownership of mealtime activity to the caregivers, resulting in an increased priority of mealtime care.

Our findings showed how *Quiet Please* created togetherness among the health professionals and thus became a path in the clinical morass. Focusing on mealtime care was important for the nurses, because they felt closer to the patients, and was a step towards a more well-adjusted and neurologically friendly environment. The intervention was described as a “game-changer” because it changed the traditional mealtime routine to a more well-adjusted and neurologically friendly environment. Other empirical studies emphasize the necessity of nutritional support; for example, stroke patients represent a vulnerable population, and support while eating is an important element of rehabilitation (Perry, Hamilton, Williams, & Jones, 2013b; Perry & McLaren, 2003). This contrasts favourably with the fact that the nursing role in nutrition has been under-recognized and poorly articulated and described (Arvanitakis, Coppens, Doughan, & Van Gossum, 2009, Kowanko, 1997; Perry, Hamilton, Williams, & Jones, 2013a). Perry et al. (2013a) argue that there has been no consensus on what constitutes the “nursing nutritional role” in relation to nutritional care. The findings in our study support this and further demonstrate how an intervention, inspired by Protected Mealtimes, redefined the nursing role in mealtime practice by combining contextual and behavioural changes. Minimizing noise, having all nurses understand the importance of aesthetics during mealtimes, and role modelling for each other when providing mealtime care were important to the patients, not only in relation to the nutritional care, but also in a broader sense because the patients, due to their neurological diagnoses, depended on a neurologically friendly environment that included consideration of environmental factors (Chaudhury, Hung, & Badger, 2013; Perry & McLaren, 2003).

Our study showed the importance of nurses including the environmental factors when caring for patients with neurological diseases. The founder of modern nursing, Florence Nightingale (1995), introduced the importance of the environment when providing mealtime care and argued for focusing beyond just the meal on the plate to the aesthetic elements when facilitating an appealing environment. Perry et al. (2013) emphasize that Kirkevold (1997) describes a specific therapeutic dimension in stroke nursing in which there are an interpretive function focused on nurses helping patients and their relatives understand the ramifications of a stroke, and a counselling function focused on the provision of emotional support. This is related to the argument of Kumlien and Axelsson (2002), who argue that the recognition of the provision of emotional support for eating difficulties is crucial, since it regards eating as a psychosocial function and enhances quality of life post-stroke (Medin, Larson, Von Arbin, Wredling, & Tham, 2010; Perry et al., 2013a). Taking this into account, provinding the intervention Quiet Please, Protected Mealtime care is not only a matter of stopping all non-acute activity and providing patients the opportunity to eat without interruptions but also a way of getting back to basic nursing as it was defined by Nightingale and providing specific nurse-led supportive care to patients hospitalized with a neurological disease (Kirkevold, 1997; Van Ort, 1995).

Our study illustrated how an intervention inspired by Protected Mealtimes provided a renewed view of the neurological patient care because the health professionals in this study felt closer to the patients and found the provision of mealtime care meaningful. However, our study also suggested that organizational structures had a high risk of conflicting with the purpose of the intervention, in which constant support for the necessity of the intervention was regarded as important. This is congruent with the arguments in the review by Perry et al. (2013) that suggest that nurses need to recognize their role and contribution in mealtime care and advocate this essential service.

Our study showed how health professionals assigned meaning in participating in an intervention. Furthermore, our study showed how the provision of Protected Mealtimes-inspired care became the story of providing fundamental neurological care. Taking a phenomenological perspective, the story about *Quiet Please* told through the perspective of the health professionals was important. Ricouer’s theory can help us understand the importance of the narrative of *Quiet Please* to the health professionals’ (working) lives in several ways.

First, analysing and interpreting the health professionals’ narratives allowed an identification and evaluation of elements that had been important at an organizational level when implementing *Quiet Please* and changing daily routines and traditions in hospital settings. In that sense, the health professionals’ stories, as conveyed in the focus-group interviews, became a narrative of how the meaning of participating in an intervention inspired by PM and intended to change mealtime practices was constructed by the health professionals.

Secondly, the narrative of *Quiet Please* also became the story of how fundamental neurological nursing should be performed. For example, the health professionals conveyed that after participating in *Quiet Please*, they had reflected on their role in the mealtime activity and prioritization of the multiple tasks that often occurred. According to Ricouer, a narrative is always more than a story of chronological events. Narratives can also be considered as abstract moments, distanced from time as a linear phenomenon. In that sense, constructing narratives in our study can be considered organizing the (professional) life experiences from a retrospective perspective but can also be considered as revealing and transforming future professional nursing identities (Kemp, 1990; Ricoeur, 1984). In relation to our study, this means that the narrative describing *Quiet Please* was important because it can be understood as a basis for ethical identity in neurological care; hence the narrative of *Quiet Please* expresses a model in which a golden standard of fundamental neurological nursing was present.

### Strength and limitations

The strengths of this study were the provision of descriptions of the intervention in our context and barriers and (pedagogical) solutions to the challenges of our intervention. As stated in the recommendations of MRC, it is important to report contextual factors because these shape and co-construct the interpretation of the intervention experience (Campbell et al., 2007; Craig et al., 2008a, 2008b). Rigorous development both validated the intervention and secured its clinical relevance in the viewpoints of the health professionals handling the intervention. Furthermore, this study illustrates how qualitative research is particularly conducive for illuminating the complexity, depth, and range of interventions, relevant to more humanized forms of care (Ludvigsen et al., 2013; Tordres, Galvin, & Holloway, 2009).

However, our study also had some methodological challenges regarding the application of Ricouer’s theory of narratives and the choice of using focus-group interviews as a method. To elucidate the phenomenon of Protected Mealtimes and to obtain nuanced, in-depth perspectives on the intervention from the health professionals, individual interviews would have strengthened our findings. Additionally, the combination of focus-group interviews and individual interviews could have provided more in-depth narratives and thereby provided more exhaustive material (Polit & Beck, 2010).

## Conclusion

We conclude that exploring the health professionals’ experiences of a mealtime intervention inspired by Protected Mealtimes was important, since they assigned meaning to the intervention on several levels. The intervention structured calmness during mealtimes and gave the health professionals a welcome moment to be in touch with their patients and their own (professional) thoughts, which was why this intervention was well-regarded by the health professionals. In this study, changing mealtime practices resembled the story of the “ugly duckling” becoming a beautiful swan. The intervention was recognized as an eye-opening event that created a sense of community in the field of neurological care in which the health professionals, and in particular the nurses, gained pride and professional identity. However, a beautiful swan needs to be nurtured to maintain its beauty. Therefore, facilitating professional support on a continuous basis and nurturing the positive elements obtained by changing mealtime practices are crucial because this study revealed that an underlying tone of rationalism and efficiency could be conflicting with the health professionals’ intentions of providing nursing and/or mealtime care.

### Relevance to clinical practice

The relevance of the study lies in its pedagogical potential to inform organizations and hospital managers about the importance of professionals’ participation in interventions changing traditional practice. This study is one of the few studies that has explored how health professionals experience interventions maintaining aesthetic elements and reveals to some extent the interplay between the surroundings and the health professionals’ experience of satisfaction with their job. It would be important to achieve deeper insight into how the health professionals assign meaning to the sensory impressions associated with the mealtime-related care. It is expected that this area can contribute with several in-depth findings that could augment the existing knowledge of the phenomena of mealtimes, and furthermore the meaning of the surroundings to the health professionals could be illuminated.
